# A structured diagnostic algorithm for patients with ARDS

**DOI:** 10.1186/s13054-023-04368-y

**Published:** 2023-03-21

**Authors:** Lieuwe Durk Jacobus Bos, Harm Jan de Grooth, Pieter Roel Tuinman

**Affiliations:** 1 Department of Intensive Care, Amsterdam UMC, Location AMC, University of Amsterdam, Amsterdam, The Netherlands; 2 Department of Intensive Care, Amsterdam UMC, Location VUMC, Vrije Universiteit Amsterdam, Amsterdam, The Netherlands

## Abstract

This article is one of ten reviews selected from the Annual Update in Intensive Care and Emergency Medicine 2023. Other selected articles can be found online at https://www.biomedcentral.com/collections/annualupdate2023. Further information about the Annual Update in Intensive Care and Emergency Medicine is available from https://link.springer.com/bookseries/8901.

## Introduction

Patients admitted to the intensive care unit (ICU) with acute respiratory failure frequently fulfil the criteria for acute respiratory distress syndrome (ARDS) [1]. The diagnosis is based on radiological, physiological, and clinical criteria described in the ‘Berlin definition’ (Table 1) [2]. Yet establishing the diagnosis of ARDS has limited treatment consequences in and of itself, as the available evidence-based interventions are mainly related to minimizing iatrogenic damage (e.g., ventilator-induced lung injury [VILI] and fluid overload) rather than the use of specific treat-ments. Whereas the intervention options for the syndrome itself are limited, adequate and timely treatment of the causal underlying condition has a major impact on the improvement of outcomes for patients with ARDS [3].

**Table 1:** Berlin definition of acute respiratory distress syndrome (ARDS) [2]

Timing	Within 1 week of risk factor^a^ *or* new/increase in respiratory symptoms
Imaging	Bilateral abnormalities not explained by pleural effusion, collapse or ‘nodules’
Origin of pulmonary edema	Insufficiently explained by cardiac failure or overload (if there is not a risk factor for ARDS, an echocardiogram should be performed)
Oxygenation	
Mild	200 < PaO_2_/FiO_2_ < 300 mmHg (26 < PaO_2_/FiO_2_ < 40 kPa) + PEEP ≥ 5 cm H_2_O
Moderate	100 < PaO_2_/FiO_2_ < 200 mmHg (13 < PaO_2_/FiO_2_ < 26 kPa) + PEEP ≥ 5cmH_2_O
Severe	PaO_2_/FiO_2_ < 100 mmHg (PaO_2_/FiO_2_ < 13 kPa) + PEEP ≥ 5 cm H_2_O

*PEEP* positive end-expiratory pressure

^a^Clinical risk factors: Pneumonia, aspiration, smoke inhalation, near drowning, sepsis, pancreati- tis, trauma, major surgery, blood transfusion (this is referred to as transfusion-related lung injury; TRALI)

The classical description of ARDS relies on the histological finding of diffuse alveolar damage secondary to another condition (one of the clinical risk factors described in Table 1) [4]. Diffuse alveolar damage is an untreatable finding and must be distinguished from a large number of diseases that also meet the ARDS syndrome definition but are treatable [5]. Table 2 provides an overview of the differential diagnoses that must be taken into account in patients suspected of having ARDS.

**Table 2:** Differential diagnoses to consider in patients with ARDS

Diffuse alveolar damage	Idiopathic; acute interstitial pneumonia
	First presentation of ILD
	Acceleration of known ILD
Cardiogenic pulmonary edema	
Infection	Bacterial pneumonia
	Viral pneumonia
	Fungal infection
	PJP pneumonia
	HSV/CMV reactivation
Interstitial lung diseases and vasculitis	Vasculitis (e.g., GPA, EGPA, and Goodpasture)
	Autoimmune disease (e.g., RA, SLE, SSc, Sjögren, antisynthetase syndrome, amyopathic (dermato) myositis and overlap syndromes)
	Medication-related: amiodarone/tyrosine kinase inhibitor/ chemotherapy / many others (www.pneumotox.com)
	Radiotherapy-associated
Malignancies	Lymphangitis carcinomatosa
	Intrapulmonary lymphoma

*ILD* interstitial lung disease, *PJP Pneumocystis jirovecii*, *HSV Herpes simplex* virus, *CMV* cyto- megalo virus, *GPA* granulomatosis with polyangiitis, *EGPA* eosinophilic granulomatosis with polyangiitis, *RA* rheumatoid artritis, *SLE* systemic lupus erythematosus, *SSc* systemic sclerosis

It should be possible to establish a definitive causal diagnosis within 7 days after onset in the vast majority of patients with ARDS. Yet, the often chaotic nature of clinical reality can lead to a delayed and haphazard search for underlying causes, especially in patients with multiple important problems.

**Fig. 1:**
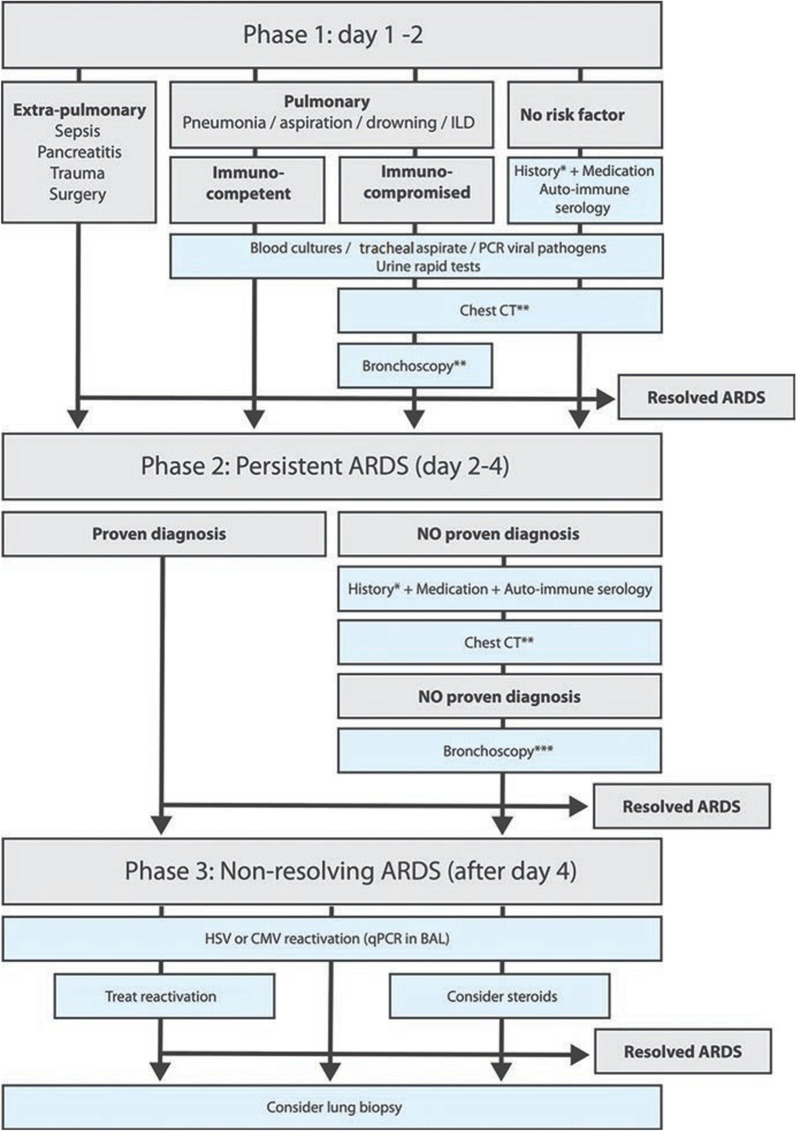
Flowchart for diagnostic steps in patient with ARDS. *Including exposure to drugs, animals, toxic fumes, vaping. **Chest computed tomography (CT) with high resolution (HR) images, preferably with an inspiratory hold. ***Send for tests described under point 6 in phase 1 in the text. *qPCR* quantitative polymerase chain reaction, *CMV* cytomegalovirus, *BAL* bronchoalveo- lar lavage, *ILD* interstitial lung disease

This narrative review aims to provide a structured approach to the diagnosis of underlying conditions in patients who fulfil the ARDS criteria according to the Berlin definition, in order to enable underlying causes to be rapidly and adequately treated. The diagnostic steps are described point by point in three phases and are summarized in a flowchart (Fig. 1). We also provide a summary of the most important uncertainties relevant to clinicians managing patients with ARDS.

With the steps and timeframe described here, we have intended to strike a balance between early and vigorous diagnostic investigations where needed and a more parsimonious approach where appropriate. Nevertheless, the authors’ experience is one rooted in academic medicine in a resource-rich environment. The details of our approach should therefore be adapted to local resources and possibilities. The most important aspect of the here described approach is not the number of laboratory investigations or imaging modalities, but rather the structuredness and timeliness of the diagnostic evaluation.

## Diagnosis of ARDS

The diagnosis of ARDS is largely based on hypoxic respiratory failure and the detection of pulmonary edema, from which hydrostatic cardiogenic pulmonary edema must be excluded [6]. It is therefore essential that false positive results are excluded as much as possible by means of non-invasive imaging. Ultrasound of the lungs is superior to chest X-ray for detecting and ruling out pleural effusion [7, 8]. The chest X-ray can give the illusion that the lung is consolidated when there is a large effusion and this can result in a false positive diagnosis, but the treatment differs. Transthoracic ultrasound of the heart can facilitate the diagnosis of acute heart failure. In general, acute heart failure is sufficient explanation for pulmonary edema and treatment should be focused on decongestion. However, a complicating factor is that cardiomyopathy due to a hyperinflammatory state can be associated with ARDS (and thus non-cardiogenic pulmonary edema) [9]. Usually, these are patients with sepsis, with a high a priori risk of ARDS who should also be diagnosed with ARDS.

### Practical steps

1. Determine that the patient meets the ARDS criteria according to the ‘Berlin definition’ and report the diagnosis of ARDS in the status (Table 1), both when the cause is evident and when the cause is unclear, as ARDS is a descriptive—and not causal—diagnosis. The presence of ARDS should trigger both a standardized set of evidence-based interventions (e.g., lung protective ventilation, restrictive fluid therapy) and, importantly, an investigation into the cause of the pulmonary injury.
2. Perform ultrasound of the lungs to rule out pulmonary effusion as the cause for the bilateral consolidations [10, 11]. Finding pleural abnormalities with lung ultrasound strongly suggests an inflammatory cause of the pulmonary edema [12].
3. Perform transthoracic cardiac ultrasound to exclude acute heart failure as a cause of pulmonary edema. Ultrasound evidence of cardiomyopathy does not exclude ARDS in an underlying condition with a high pre-probability of ARDS (such as sepsis) and in this case ARDS should be diagnosed despite the contribution of acute heart failure to the onset of pulmonary edema.

### Uncertainties

- Patients treated with high-flow nasal oxygenation do not meet the Berlin definition of ARDS [13], and there is considerable uncertainty about optimal lung-protective strategies in these patients. Yet the structured investigation into the cause of lung injury (outlined below) should not be delayed merely because the formal definition has not been met.
- Lung ultrasound may be used for the diagnosis of bilateral opacities and might be used to diagnose non-cardiogenic pulmonary edema [14]. There is uncertainty about the best algorithmic approach to ARDS diagnosis based on lung ultra- sound and the diagnostic test characteristics.
- Cardiac ultrasound can provide diagnostic evidence in favor of heart failure, but it is unclear what cutoffs truly exclude ARDS as cause.

## First Phase of Evaluation (Days 1 and 2)

Extra-pulmonary and pulmonary risk factors for ARDS need to be identified as soon as possible. When an evident extra-pulmonary cause for ARDS, such as septic shock, is present, timely treatment of the underlying cause determines the patient’s prognosis [15]. Patients with community-acquired pneumonia *and* ARDS are indis- tinguishable from patients in the ICU with community-acquired pneumonia without ARDS in terms of epidemiological data, microbiological results, and outcome, and likely require the same treatment [16]. As opportunistic infections can present with specific radiological patterns that cannot be appreciated on chest X-ray, a chest computed tomography (CT) scan should be performed in patients with increased a priori risk for such infections (see practical steps) [17]. If no risk factor can be iden- tified, there is an increased probability of an underlying systemic disease or drug related cause [5]. Further information for this should be obtained through history, physical examination, autoimmune serology and chest CT. The most important serological tests are: extractable nuclear antigens (ENA), anti-nuclear antibodies (ANA), anti-neutrophil cytoplasmic antibodies (ANCA), myositis blot, anti-cyclic citrulline peptide antibody (aCCP) and rheumatoid factor (RF). When hemoptysis and/or acute renal insufficiency with (microscopic) hematuria are present, anti- glomerular basal membrane (aGBM) levels should also be obtained [18].

### Practical steps

1. Determine whether there is an extra-pulmonary or a pulmonary cause for ARDS.
2. If an extra-pulmonary cause seems very likely, no search for an underlying pul- monary disease is required in the first phase. The underlying condition must be treated.
3. In case of pneumonia in a patient with a normal immune system, no invasive diagnostic tests need to be performed in the first 48 h. Required diagnostics are sputum culture, respiratory mutiplex polymerase chain reaction (PCR), antigen tests, and blood cultures.
4. An immunocompromised patient can be defined by one or more of the following criteria:
  - Severe neutropenia (absolute neutrophil count < 500/μl) or prolonged lym- phopenia (absolute lymphocyte count < 1000/μl for > 7 days)
  - Hematological malignancy
  - Long-term steroid exposure (≥ 20 mg/day prednisone equivalent for more than 2 weeks)
  - Status after organ transplantation
  - Monoclonal antibodies or other anti-inflammation immunosuppressive medications (e.g., azathioprine, mycophenolate mofetil, methotrexate)
  - Known immunodeficiency such as human immunodeficiency virus (HIV) with CD4 + cell count of less than 200/mm^3^.
5. In an immunocompromised patient with suspected pneumonia, a chest CT should be performed to evaluate the radiological pattern of lung involvement.
6. In an immunocompromised patient with suspected pneumonia, bronchoscopy should be performed with bronchoalveolar lavage (BAL) for bacterial and fungal culture, galactomannan and targeted PCRs for respiratory pathogens, including but not limited to respiratory viruses, *Aspergillus*, *Pneumocystis jirovecii* (PJP), cytomegalovirus (CMV) and Herpes simplex virus (HSV)—depending on the pattern on chest CT.
  - For specific radiological images, additional microbiological investigation should be considered, for example *Nocardia* or *Cryptococcus* in the context of nodular abnormalities.
  - One fraction should be sent for cytology, especially if eosinophilic pneumo- nia or malignancy is in the differential diagnosis.
  - If diffuse alveolar hemorrhage is considered, gradual rinsing with saline should be performed.
7. If no risk factors for ARDS are present an alternative diagnosis should be inves- tigated through a complete re-evaluation of history and complete physical examination.
  - Pay attention specifically to systemic diseases (Table 2).
  - If clinical signs and/or symptoms consistent with a systemic disease are found, low-threshold autoimmune serology should be used. Also determine the creatinine kinase and urine sediment on dysmorphic erythrocytes. If indicated, additional scleroderma immunoassay, complement, lupus antico- agulant test, anti-cardiolipins and B2 glycoprotein1.
  - If diffuse alveolar hemorrhage is considered, in the context of vasculitis or not, the anti-GBM must also be determined.
8. The medication list should be systematically reviewed to identify and discon- tinue potentially pulmonary toxic medications (see www.pneumotox.com).
9. A chest CT should be considered based on the diagnostic information obtained in the previous steps or if the patient deteriorates within 48 h.

### Uncertainties

- Immunosuppressed patients are frequently grouped together in critical care research and it is largely unclear how different types of immunosuppression (e.g., predominant granulocyte function, T-cell or B-cell immunity) influence the risks for opportunistic infections in critically ill patients [19].
- The microbial diagnosis of opportunistic infections has shifted from traditional diagnostic techniques to PCR-based technology. There is considerable uncer- tainty surrounding the best cut-offs for these diagnostic tests. For example, CMV and HSV pneumonitis are nowadays frequently diagnosed using PCR, but little evidence on optimal cut-offs exists, which could result in over-diagnosis and over-treatment [20–22].
- With the increase in polypharmacy and increased use of novel drugs, there is more risk for drug-related pulmonary toxicity. Although pulmonary toxicity has been described for many of the frequently used drugs, it is very difficult to reach a definitive diagnosis as no diagnostic tests are available [23, 24].

## Second phase of evaluation (Days 3–5)

A considerable proportion of patients will improve during the first phase of evalua- tion and in other patients it will be possible to establish a definitive causal diagnosis [25]. If after 2 days no causative agent of pneumonia has been demonstrated, and if the clinical condition of the patient does not improve, it is important to reconsider the diagnosis. To not miss any mimicking conditions, the same approach is followed as in patients without a risk factor at presentation including but not limited to a detailed history, physical examination, and autoimmune serology.

Performing a chest CT scan can help distinguish between opportunistic infections and interstitial lung diseases [17, 18, 26]. A bronchoscopy with lavage is a reliable method to take microbiological cultures from the lower respiratory tract [18], espe- cially if PCR analyses are performed for viruses and opportunistic pathogens such as *Pneumocystis* and *Aspergillus*. Bronchoscopy can result in loss of pressure from the ventilation system and thus to collapse of previously opened parts of the lung. However, multiple studies suggest that bronchoscopy with lavage is safe in intubated patients with ARDS provided that it follows the prevailing guidelines [27–29].

### Practical steps

1. Determine whether the risk factor for ARDS has been proven, for example because a pathogen was detected in the context of an infection.
2. If the risk factor for ARDS has been proven, treatment should be continued and possibly optimized. In this case, there is no need to look further for alternative diagnoses, unless there are clear diagnostic clues pointing towards a second cause for lung injury.
3. If there was a suspected cause, but it remains unproven after day 2, a complete re-evaluation of autoimmune disorders, toxic medications, and chest CT should be performed in patients in whom this was not performed at an earlier stage (see Sects. "Conclusion"–8 of phase 1).
4. The chest CT findings should prompt consideration of bronchoscopy with lavage (see Sect. "Conclusion" of phase 1).

## Third phase of evaluation (Days 6–7)

If ARDS persists for more than 5 days after diagnosis it is considered as ‘non-resolving’ and the risk of fibrosis formation is considerable if the cause for ARDS remains untreated [3, 30]. There are several studies that show that reactivation of HSV and CMV is frequent in non-resolving ARDS [31–33]. No randomized trials have been conducted on whether or not to treat HSV reactivations, but observational studies suggest independent excess mortality in the HSV and CMV group. Due to the lack of other treatable conditions in this patient group, it is therefore advisable to treat reactivation [18].

There is conflicting evidence from several randomized trials of corticosteroid treatment for non-resolving ARDS. There appears to be a positive effect on ventilation duration and possibly on mortality if treatment is started early in the non-resolving phase, e.g., before day 14 [34–36]. Apart from hyperglycemias, relatively few adverse reactions have been described [37].

In patients in whom ARDS persists and in whom the underlying cause has not been confirmed despite all the above steps, a lung biopsy should be considered. The risks of an open lung biopsy (< 10% serious complications, < 1% lethal complications [38]) must be weighed against the substantial burden of ongoing ICU treatment without a clear diagnosis. Open lung biopsy provided a specific diagnosis in about 75% of cases and led to an adjustment in medication in about one in three patients [38]. In addition, findings other than diffuse alveolar damage are associated with change in therapy and better outcome [32, 39–42]. Prolonged ventilation in the context of non-resolving ARDS without a diagnosis has a very poor prognosis and can result in unnecessarily prolonged ICU treatment with all the adverse consequences that this entails. All in all, this results in the recommendation to consider an open lung biopsy in all patients with non-resolving ARDS without a proven risk factor [18]. Traditionally, a surgical open lung biopsy is obtained by thoracoscopy, but there are new developments in the field of bronchoscopically obtained cryobiopsies that can be considered as an alternative [43].

### Practical steps

1. Consider HSV or CMV reactivation as a contributing factor for lung inflammation in non-resolving ARDS. A bronchoscopy with BAL should then be performed for quantitative PCR of these viruses.
2. Consider high dose corticosteroid treatment in patients with non-resolving ARDS. Treatment early in the non-resolving phase, before day 14, is associated with a better outcome and is therefore recommended.
3. If ARDS persists on days 5–7 and the diagnosis is not confirmed despite all the above steps, it is recommended to discuss performing a lung biopsy in a mul- tidisciplinary meeting. It is of utmost importance to provide the pathologists with all available clinical information to come to the best possible diagnosis (Table 3).

**Table 3:** Diagnostic patterns to consider for open lung biopsy in non-resolving ARDS

Infection
*Connective tissue disease*
Drug reaction
*Eosinophilic pneumonia*
*Blood suggestive of vasculitis*
Foreign material (inhaled, aspirated, injected)
*Scarring*
Hypersensitivity pneumonitis

### Uncertainties

- HSV and CMV reactivation could be a marker of severity of disease or an etio- logical factor hampering the resolution of ARDS. Currently, it remains unclear whether treatment of HSV or CMV reactivation actually improves clinical out- comes [22].
- There is considerable variation in the use of high dose steroids for the treat- ment of non-resolving ARDS. One randomized controlled trial showed steroid use was associated with shortened duration of invasive mechanical ventila- tion, but had no effect on mortality [44]. Furthermore, the dosage and duration of treatment is open for debate, although some guidance based on pharmaco- logical principles is available [45].
- The available literature on the diagnostic and therapeutic consequences of open lung biopsy is largely based on data acquired before the widespread implementa- tion of molecular testing for pathogens. Current diagnostic test characteristics are therefore uncertain.

## Conclusion

Establishing the underlying cause of ARDS is of great importance as adequate treat- ment of this cause improves outcome. The proposed structured diagnostic algorithm helps clinicians to systematically evaluate patients with ARDS and to decrease time to diagnosis and thereby start of adequate treatment.

## Data Availability

Not applicable.
